# Molecular and *in-silico* analysis of single nucleotide polymorphism targeting human *TP53 *gene exon 5-8 in Sudanese esophageal cancer patients

**DOI:** 10.12688/f1000research.15534.1

**Published:** 2018-11-02

**Authors:** Rihab M. Elfaki, Mohammed S. Abdelaziz, Hisham N. Altayb, Munsoor M. Munsoor, Ahmed A. Gameel

**Affiliations:** 1 Department of Histopathology and Cytology, Sudan University of Science and Technology, Khartoum, Khartoum, 11111, Sudan; 2 Department of Microbiology, Sudan University of Science and Technology, Khartoum, Khartoum, 11111, Sudan; 3 Department of Heamatology, Sudan University of Science and Technology, Khartoum, Khartoum, 11111, Sudan; 4 Department of Pathology, University of Khartoum, Khartoum, Khartoum, 11111, Sudan

**Keywords:** Protein 53, SNPs, in silico Analysis, Esophageal cancer, Sudan.

## Abstract

**Background:** The protein product of the normal *TP53* gene performs an essential function in cell cycle control and tumor suppression, and the mutation of a *TP53* gene is an essential step in the development of many cancers. Despite the reported association of *TP53* gene mutations with many human cancers, the comprehensive computational analysis of single nucleotide polymorphisms (SNPs), and their functional impacts, still remains rare.

**Methods:** In this study DNA were extracted from formalin fixed paraffin embedded samples followed by the conventional polymerase chain reaction and DNA sequencing. Computational analysis was performed using different algorithms to screen for deleterious SNPs.

**Results:** The results demonstrate that there are synonymous SNPs (sSNPs) and non-synonymous SNPs (nsSNPs) in the *TP53* gene that may be deleterious to p53 structure and function. Additionally, *TP53* gene mutations were found in 40% of samples. Six out of ten of *TP53* gene mutations occurred in exon 5, two mutation in exon 6 and other two were present in exon 8. Only one SNP in position E298Q was predicted to have a neutral effect and other SNPs were predicted to be disease related according to Mutation Taster software. A total of 37.2% of squamous cell carcinoma (SCC) samples were found to be mutated, 87.5% of them exist in exon 5, 12.5% in exon 6 and 6.3% in exon 8, whereas adenocarcinoma (AC) achieved a higher rate of mutation (57.1%) with 100% exon 5 involvement.

**Conclusions:** Mutation of *TP53* exon 5 in esophageal cancer patients were the most frequent. Genomic results have identified a higher *TP53* mutation rate in esophageal AC in contrast to SCC.

## Introduction

Esophageal cancer is considered one of the eight most common cancers throughout the world, and is also one of the most fatal cancers, taking into account its aggressiveness and reduced survival rate. Because of its poor prognosis with 5-year survival rates ranging between 10–13%, it ranks sixth among all cancers in mortality rate ^1– 4^.

Knockout of *TP53* in mice leads to the development of different tumors, including lymphomas, sarcomas adenocarcinoma and benign tumors such as hemangioma, before they reach 6 month of age ^5^.

*TP53* gene encodes a tumor suppressor protein which plays an important role inside the cell especially in DNA transcription and repair, senescence, apoptosis, tumor suppression, treatment response and also the response to changes in metabolism ^6, 7^. Protein domains represent independently folding units of protein with a size between 40 to 200 amino acids. Human p53 protein contains three domains; transcriptional activation, DNA binding, and oligomerization domains. These domains are edged by a connecting region. A proline-rich region links the transcriptional activation and DNA binding domains, a second proline-rich region links the DNA binding and oligomerization domains and a basic region form the C-terminus of the protein ^8^. The evolutionarily highly conserved core domain (amino acids ~100 to ~300) is involved in sequence-specific binding to promoters of p53-regulated genes ^9^.

Single nucleotide polymorphisms (SNP) are a significant type of genetic variation commonly detected in the human genome. SNPs occur in non-coding regions as well as in coding regions of the genome ^10, 11^. A total of 336,845,724 SNPs have been identified in humans so far, and have been deposited in NCBI dbSNP. The human *TP53* gene has 3115 identified SNPs. SNPs arise in coding regions may cause an amino acid change in the corresponding protein and in such case it is called as non-synonymous SNP (nsSNP) or may not change the amino acid and here it is called a synonymous SNP (sSNP); these nsSNPs change the protein structure and hence its function, causing a specific disease ^12, 13^.

Recently a number of articles have demonstrated the association of SNPs in the *TP53* gene with different cancer types, but *in silico* analysis has not yet been discussed on the functional, interactional and structural aspects of different types of SNPs in this gene. In the current study, we used different bioinformatics prediction tools and databases for analysis of these SNPs in *TP53* gene. As a significant number of mutations have an impact on protein stability and interactions with the corresponding proteins, we also offered a structural model of the mutant protein. Here in this study the main objective is to detect mutations of *TP53* gene focusing on exons 5 to 8 among esophageal cancer patients as these has been reported as the most mutated exons in this gene ^14^.

## Methods

### Sampling

Sections of 30–40 µm thickness from 50 formalin fixed paraffin embedded (FFPE) tissue samples were obtained from esophageal cancer patients representing different hospitals and clinics in Khartoum State, Sudan, from July 2013 to June 2017. All patients have been previously diagnosed with squamous cell carcinoma (SCC) and adenocarcinoma (AC).

### DNA extraction

Genomic DNA for PCR analysis was extracted from FFPE tissue blocks. Using commercial DNA extraction kits for fast isolation of genomic DNA from FFPE samples as per manufacturer’s instructions. Extraction procedure is based on combination of an efficient lysis step with a subsequent binding of genomic DNA on a Spin Filter surface followed by washing of the bound DNA and finally eluting of the DNA (845-BP-0020250, black PREP FFPE DNA Kit, Analytik Jena Company).

### PCR amplification

For amplification of exon 5, 6,7 and 8 of *TP53* gene, four pairs of primers (catalogue numbers: 171002-009_D5, 171002-009_D6, 171002-009_D7, 171002-009_D8, 171002-009_D9, 171002-009_D10, 171002-009_D11, 171002-009_D12; Macrogen, Korea) were used ^15, 16^ ( Table 1). A total of 2–5 µl of genomic DNA, 0.5 µl forward and 0.5 µl reverse primer and 25 μl double distilled water (DDW) was combined to make up the final reaction volume. The mixture was amplified using Heal force thermal cycler (Model No: K960) with the following amplification conditions; 95°C for 5 min, followed by 37 cycles at 95°C for 45 sec, primer-specific annealing temperature for 45 sec, 72°C for 45 sec and a final extension at 72°C for 5 min. 5 μl of the PCR products were applied on 2 % agarose gel and remaining PCR products were sequenced by BGI company (China).

**Table 1.** Primer sets specifications.

Exon	Sequence	A. Temp.	P. Size
**Exon 5:**	**Forward:** 5'-TAC TCC CCT GCC CTC AAC AA-3'	59.7°C	184
	**Reverse:** 5'-CAT CGC TAT CTG AGC AGC GC-3'		
**Exon 6:**	**Forward:**5’-TTG CTC TTA GGT CTG GCC CC-3’	58.2°C	128
	**Reverse:** 5’-CAG ACC TCA GGC GGC TCA TA-3’		
**Exon 7:**	**Forward:**5’-TAG GTT GGC TCT GAC TGT ACC-3’	59.3°C	117
	**Reverse:** 5’-TGA CCT GGA GTC TTC CAG TGT-3’		
**Exon 8:**	**Forward:**5’-AGT GGT AAT CTA CTG GGA CGG-3’	53.8°C	141
	**Reverse:**5’-ACC TCG CTT AGT GCT CCC TG-3’		
A; annealing, P; product			

### Dataset collection

The SNP information SNP ID, mRNA accession number NM_000546, and Protein accession number NP_000537 of the human *TP53* gene used in our computational analysis were retrieved from the National Center for Biotechnology Information (NCBI) database and catalogue of somatic mutation in cancer (COSMIC) database (TP53_ENST00000269305). The nucleotide and amino acid sequence of the p53 protein were obtained and investigated using nucleotide ( NG_017013), Gene ( Gene ID: 7157) database NCBI and UniProt database ( P04637).

### Data analysis using different bioinformatics tools

***Codon code aligner*.** Sequences were assembled into contigs end clipped and edited using Codon Code Aligner software version 8.0.1 (Dedham, MA, USA). Sequence data are available at GenBank under accession numbers MH366303 to MH366483 ^17^.

***SIFT Program*.** SIFT (Sorting Intolerant from Tolerant) tool uses sequence homology to calculate the probability of affecting protein function in case of amino acid change. It uses the concept of evolutionarily conserved regions which is less tolerant to mutations, and therefore amino acid change or frame shift mutations in these regions are expected to affect protein function the most. SIFT tool works by introducing a query protein into SIFT program to be searched against protein database aligned with homologous protein sequences. Then the program calculates SIFT score based on amino acid changes in that position. A SIFT score ranges from 0 to 1. Score less than 0.05 is predicted to affect protein function and considered functionally deleterious, whereas any score more than or equal to 0.05 represents a neutral substitution ^18– 20^.

***PolyPhen -2*.** PolyPhen-2 (Polymorphism Phenotyping version 2) is a structural and functional predicting tool that predicts the effect of an amino acid change on protein characteristics based on SNPs functional annotations, protein structural properties with sequence annotation, and finally predict if the coding non-synonymous SNPs are considered damaging or not ^21, 22^.

PolyPhen-2 workflow requires protein sequence, mutational position, and substitution. The PolyPhen output is represented with a score that ranges from 0 to 1, with zero score indicating a neutral effect of amino acid substitution on protein function and a higher score representing a mutation that is more likely to be damaging ^23^.

***I-Mutant 3.0*.** I-Mutant 3.0 is a support vector machine (SVM) based tool, which was used to calculate the stability changes of specific SNP upon protein sequence. Information of wild and mutated residue, protein sequence, temperature, and pH was used as input parameters to this server, and finally, the outputs reports if a point mutation is stable or not. The program categorizes the prediction into: neutral mutation (DDG = 0.5 kcal/mol), large decrease of stability (0.5 kcal/mol). The output is a free Gibbs energy change value (ΔΔG) of protein before and after mutation ^24– 27^.

***PhD-SNP*.** PhD-SNP (Predictor of Human Deleterious Single Nucleotide Polymorphisms) software is a prediction tool that predicts disease association of nsSNP by dividing those SNPs into disease-related or neutral polymorphism based on a score ranged from (0-1); SNPs with a score above 0.5 are considered disease associated according to the program algorithm. PhD-SNP outputs depend on a number of sequences aligned, conservation index of SNP position, frequencies of wild and mutant residues ^19, 20, 28^.

***Project HOPE*.** Structural and biochemical analysis for mutations was accomplished using Project HOPE is a web-server used to give a comprehensive report on the effect of the specific mutation on the 3D structure of the native protein and the variant model using different software and sources. The user can submit a protein sequence or an accession number of specific protein after specifying the wild-type residue and the new mutant form to create the report ^29, 30^.

***Mutation Taster*.** Mutation Taster calculates the pathogenic consequences of variations in DNA sequence. It predicts the functional impact of amino acid alterations, intronic and synonymous substitutions, in addition to INDEL mutations and variants covering intron-exon connection region. Mutation Taster prediction system divides alterations as; Disease-causing: which is probably deleterious, Disease-causing automatic: the alteration here is known to be deleterious, Polymorphism: probably harmless alteration and polymorphism automatic: known to be harmless ^31– 33^.

***FATHMM*.** FATHMM (Functional Analysis Through Hidden Markov Models) is a web-server predicts the functional significances of both coding and non-coding variants. We selected the cancer option to display predictions that can distinguish between cancer-promoting/driver mutations and other germline polymorphisms. It uses a default prediction threshold of -0.75. Predictions with scores less than this indicate that the mutation is potentially cancer associated ^34^.

## Results

### Results of *TP53* gene mutations in esophageal carcinomas

Esophageal squamous cell carcinoma cases represent 43 (86%) of all cases, whereas adenocarcinoma made up 7 cases (14%). Mutation analysis results demonstrate a higher *TP53* mutation rate in esophageal adenocarcinoma compared to squamous cell carcinoma. This were illustrated in ( Table 2) in the results section which describe Histopathological diagnosis, mutational status and exons affected in esophageal cancer patients.

**Table 2.** Histopathological diagnosis, mutational status and exons affected in esophageal cancer patients.

No.	Tumor type	mutational status	Mutated exons
17	Squamous Cell Carcinoma	**-**	**-**
80	Squamous Cell Carcinoma	**-**	**-**
117	Squamous Cell Carcinoma	**-**	**-**
54	Squamous Cell Carcinoma	**-**	**-**
10	Squamous Cell Carcinoma	**+**	**5**
34	Squamous Cell Carcinoma	**+**	**6**
57	Squamous Cell Carcinoma	**-**	**-**
58	Squamous Cell Carcinoma	**-**	**-**
59	Squamous Cell Carcinoma	**+**	**5**
28	Squamous Cell Carcinoma	**+**	**5**
48	Squamous Cell Carcinoma	**+**	**5**
38	Squamous Cell Carcinoma	**-**	**-**
120	Squamous Cell Carcinoma	**+**	**5**
16	Squamous Cell Carcinoma	**+**	**5**
24	Squamous Cell Carcinoma	**+**	**5**
7	Squamous Cell Carcinoma	**+**	**5**
9	Squamous Cell Carcinoma	**+**	**5,6**
8	Squamous Cell Carcinoma	**+**	**5**
2	Squamous Cell Carcinoma	**-**	**-**
67	Squamous Cell Carcinoma	**-**	**-**
114	Squamous Cell Carcinoma	**-**	**-**
98	Squamous Cell Carcinoma	**-**	**-**
23	Squamous Cell Carcinoma	**+**	**5**
1	Squamous Cell Carcinoma	**+**	**5**
52	Squamous Cell Carcinoma	**-**	**-**
82	Squamous Cell Carcinoma	**-**	**-**
43	Squamous Cell Carcinoma	**-**	**-**
29	Squamous Cell Carcinoma	**-**	**-**
62	Squamous Cell Carcinoma	**-**	**-**
49	Squamous Cell Carcinoma	**+**	**8**
36	Squamous Cell Carcinoma	**-**	**-**
112	Squamous Cell Carcinoma	**-**	**-**
89	Squamous Cell Carcinoma	**-**	**-**
74	Squamous Cell Carcinoma	**-**	**-**
87	Squamous Cell Carcinoma	**-**	**-**
88	Squamous Cell Carcinoma	**-**	**-**
116	Squamous Cell Carcinoma	**-**	**-**
115	Squamous Cell Carcinoma	**+**	**5**
110	Squamous Cell Carcinoma	**-**	**-**
93	Squamous Cell Carcinoma	**-**	**-**
119	Squamous Cell Carcinoma	**-**	**-**
97	Squamous Cell Carcinoma	**-**	**-**
26	Squamous Cell Carcinoma	**+**	**5**
103	Adenocarcinoma	**-**	**-**
56	Adenocarcinoma	**-**	**-**
19	Adenocarcinoma	**+**	**5**
12	Adenocarcinoma	**+**	**5**
6	Adenocarcinoma	**+**	**5**
65	Adenocarcinoma	**-**	**-**
50	Adenocarcinoma	**+**	**5**

**Figure 1.**
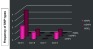
Distribution of TP53 coding synonymous SNPs (sSNPs), coding non-synonymous SNPs (nsSNPs) and INDEL through exon 5–8 in P53 gene.

Distribution of TP53 coding synonymous SNPs (sSNPs), coding non-synonymous SNPs (nsSNPs) and INDEL through exon 5-8 in P53 gene were represented by ( Figure 1).

*TP53* gene SNPs were found in 20 out of 50 sample of esophageal carcinomas (40%). Six out of ten SNPs (60%) were missense SNPs leading to amino acid substitution, four SNPs (40%) were silent mutations without any amino acid change. Six out of ten (60%) *TP53* SNPs occurred in exon 5 at the following codons 160, 161 (twice), 163, 164, 175. Three of them were missense and three were silent. Two SNPs (20%) were located in exon 6 at codon 215 (missense) and 222 (silent). Two SNPs (20%) were present in exon 8 at codon 298 (missense) and 305 (silent).

15 out of 43 (34.9%) SSC samples were found to be mutated, 13/15 (86.7%) of them existed in exon 5, 2/15 (13.3 %) in exon 6 and 1/15 (6.7%) in exon 8. Whereas adenocarcinoma had a higher rate of mutations 4/7 (57.1%) with 100% of SNPs occurring in exon 5.

**Figure 2.**
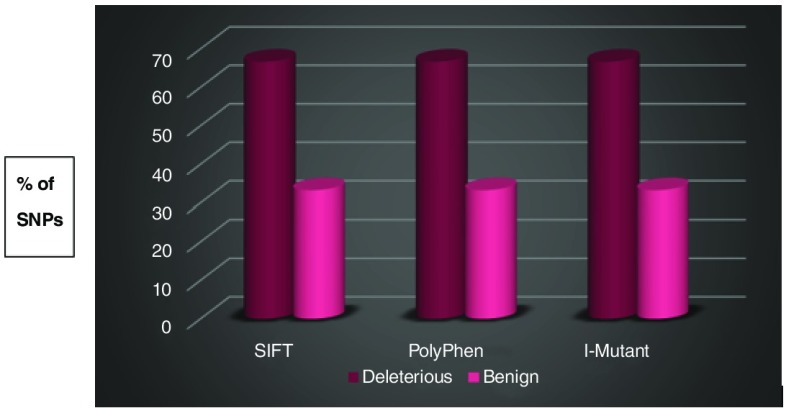
Distribution of deleterious and benign non-synonymous single nucleotide polymorphisms (nsSNPs) by Sorting Intolerant From Tolerant (SIFT), Polymorphism Phenotyping v2 (PolyPhen), and I-Mutant Suite. The magenta cylindrical bar indicates the percentage of nsSNPs that were found to be deleterious by SIFT, damaging (Possibly/Probably) by PolyPhen, and largely unstable by I-Mutant Suite. The pink cylinder indicates the percentage of nsSNPs that were found to be tolerated by SIFT, benign by PolyPhen, and largely stable/neutral by I-Mutant Suite.

The percentage of deleterious nsSNPs predicted by SIFT and PolyPhen was 66.7% ( Figure 2), those SNPs were A161D, K164E, R175P and S215N according to SIFT and PolyPhen ( Table 3– Table 4). I-Mutant suite also give the same percentage for deleterious nsSNPs which is 66.7% ( Table 5) but in case of using I-Mutant suite the predicted SNPs to be deleterious were, A161D, R175P, S215N and M160V ( Table 6). The PhD-SNP report defines 5/6 (83.3%) of nsSNPs as disease related polymorphism ( Table 7).

**Table 3.** List of variants that were analyzed using SIFT algorithm.

Protein ID	Nucleotide change	AA Substitution	Prediction	Score	Median Info
ENSP00000269305	A/G	M160V	tolerant	0.27	2.76
ENSP00000269305	C/A	A161D	damaging	0.01	2.76
ENSP00000269305	A/G	K164E	damaging	0.00	2.76
ENSP00000269305	G/C	R175P	damaging	0.00	2.76
ENSP00000269305	G/A	S215N	damaging	0.00	2.76
ENSP00000269305	G/C	E298Q	tolerant	0.39	2.75

SIFT: Sorting Intolerant From Tolerant. SIFT score ≤ 0.05 considered damaging. SIFT Tolerance Index: Ranges from 0 to 1. AA: Amino acid

**Table 4.** Prediction of functionally significant nsSNPs by PolyPhen 2.0 algorithm.

COSMIC ID	Amino acid change	Prediction	Score
COSM44328	M160V	Benign	0.177
COSM44391	A161D	Probably damaging	1.000
COSM10762	K164E	Probably damaging	0.997
COSM45416	R175P	Probably damaging	1.000
COSM44093	S215N	Probably damaging	1.000
COSM45938	E298Q	Benign	0.003

nsSNPs: Non-synonymous single nucleotide polymorphisms, PolyPhen: Polymorphism Phenotyping v2.

**Table 5.** The Prediction Results of nsSNPs of human *TP53* Using SIFT, PolyPhen and I-Mutant 3.0 algorithms.

Prediction Result	SIFT		PolyPhen		I Mutant 3.0	
	No. of nsSNPs	%	No. of nsSNPs	%	No. of nsSNPs	%
Deleterious	4	66.7 %	4	66.7 %	4	66.7 %
Tolerated	2	33.3 %	2	33.3 %	2	33.3 %
Total	6	100%	6	100%	6	100%

SIFT: Sorting Intolerant From Tolerant. The amino acid substitution is predicted deleterious if the score is <= 0.05, and tolerated if the score is > 0.05.

**Table 6.** Prediction of nsSNPs stability status by I Mutant 2.0 algorithm.

SNP ID’S	Amino acid change	SVM3 Prediction Effect	RI	∆∆G (kcal/mol)
COSM44328	M160V	Large Decrease	5	-0.60
COSM44391	A161D	Large Decrease	3	-0.52
COSM10762	K164E	Neutral	3	-0.08
COSM45416	R175P	Large Decrease	0	-0.57
COSM44093	S215N	Large Decrease	3	0.58
COSM45938	E298Q	Neutral	1	-0.25

I-mutant RI (Reliability Index): 0–10, where 0 is the lowest reliability and 10 is the highest reliability.

**Table 7.** Prediction of functionally significant nsSNPs, by PhD-SNP.

Mutation	PhD-SNP	
	Prediction	RI
K164E	Disease-related polymorphism	7
A161D	Disease-related polymorphism	9
M160V	Disease-related polymorphism	6
S215N	Disease-related polymorphism	5
E298Q	Neutral	5
R175P	Disease-related polymorphism	8

The results reveal that SNPs in positions E298Q were predicted to be a neutral polymorphism which represent 10% of all mutation detected. All other SNPs M160V, A161D, A161A, Y163Y, K164E, R175P, S215N, P222P, and K305K representing 90% of all SNPs were predicted to be disease related according to MutationTaster software ( Table 8).

**Table 8.** Summary of SNP Characteristics identified in *TP53* gene exon 5, 6 and 8.

Localization	Patient affected	alleles	AA Change	Position	SNP ID	Type	Significance *
**Exon 5**	14	A/G	K164E	7675122	rs879254249	Missense	Disease causing
**Exon 5**	1	C/A	A161D	7675130	rs1064795691	Missense	Disease causing
**Exon 5**	9	A/G	M160V	7675134	rs377274728	Missense	Disease causing
**Exon 5**	2	C/T	Y163Y	7675123	COSM44391	Coding-synonymous	Disease causing
**Exon 5**	1	G/C	R175P	7675088	COSM45416	Missense	Disease causing
**Exon 5**	1	C/T	A161A	7675129	COSM44119	Coding-synonymous	Disease causing
**Exon 6**	1	G/A	S215N	7674887	rs587782177	Missense	Disease causing
**Exon 6**	1	G/C	P222P	7674865	COSM43924	Coding-synonymous	Disease causing
**Exon 8**	1	A/T	E298Q	7673728	Novel	Missense	Polymorphism
**Exon 8**	1	G/A	K305K	7673705	COSM46382	Coding-synonymous	Disease causing

*according to MutationTaster.

Frequency of SNPs among different samples was shown in details in ( Table 9). FATHMM server; cancer association predictions result of the non-synonymous changes found in TP53 gene exon 5-8 were demonstrated in ( Table 10).

**Table 9.** SNPs information and frequency among different samples.

Alleles	Position in chromosome	Exon	AA change	Samples with the same SNP	SNP frequency Percentage
A/G	7675134	5	M160V	9 out of 50	18 %
C/A	7675130	5	A161D	1 out of 50	2 %
A/G	7675122	5	K164E	14 out of 50	28 %
G/C	7675088	5	R175P	1 out of 50	2 %
G/A	7674887	6	S215N	1 out of 50	2 %
G/C	7673728	8	E298Q	1 out of 50	2 %

**Table 10.** FATHMM server; cancer association predictions result of the non-synonymous changes found in *TP53* gene exon 5–8.

COSMIC ID	Amino acid change	Prediction	Score
COSM44328	M160V	CANCER	-9.03
COSM44391	A161D	CANCER	-9.34
COSM10762	K164E	CANCER	-9.12
COSM45416	R175P	CANCER	-9.93
COSM44093	S215N	CANCER	-9.58
COSM45938	E298Q	CANCER	-8.11

**Figure 3.**
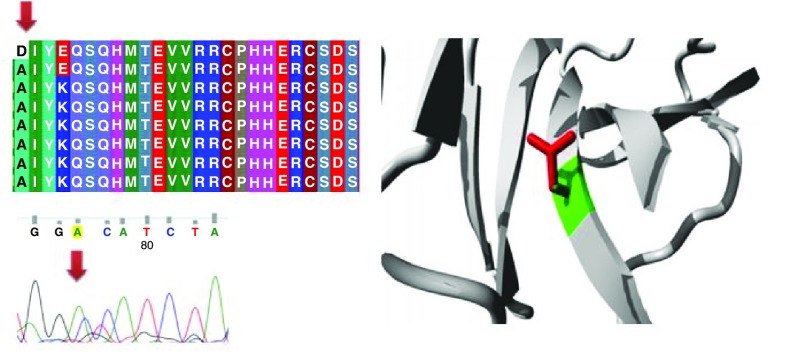
Mutation of alanine into aspartic acid at position 161.

Different mutations with their position, wild type and mutant form in addition to alignment and chromatogram were illustrated in ( Figure 3– Figure 8).

**Figure 4.**
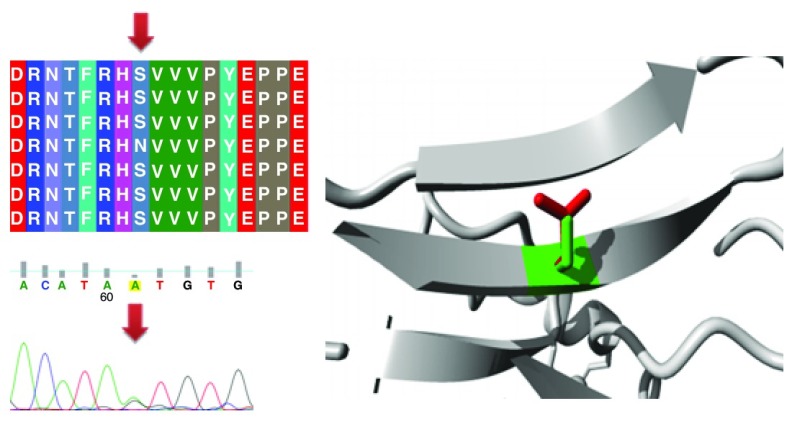
Mutation of a serine into asparagine at position 215.

**Figure 5.**
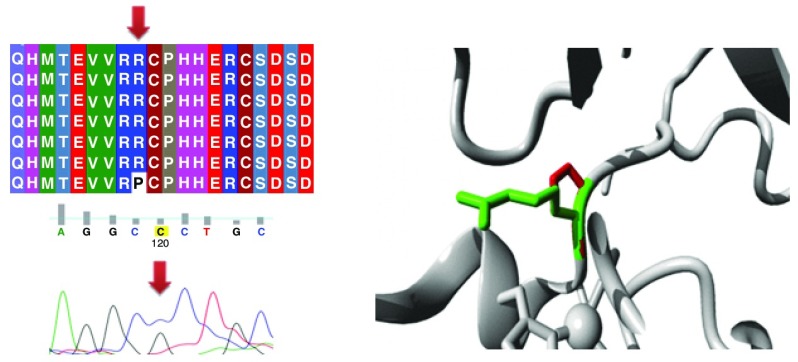
Mutation of arginine into proline at position 175.

**Figure 6.**
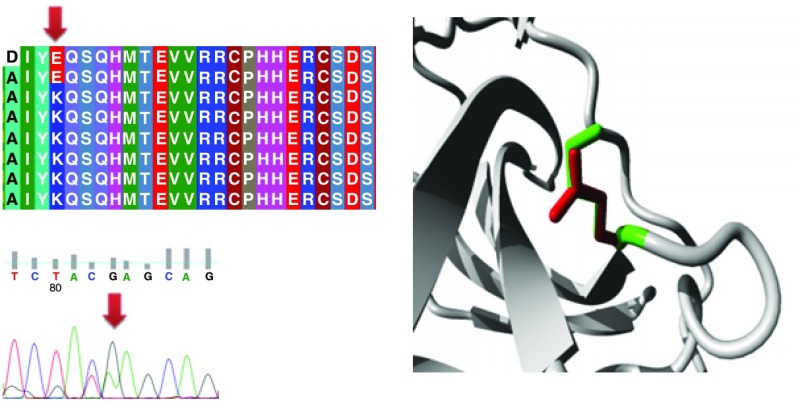
Mutation of lysine into glutamic acid at position 164.

**Figure 7.**
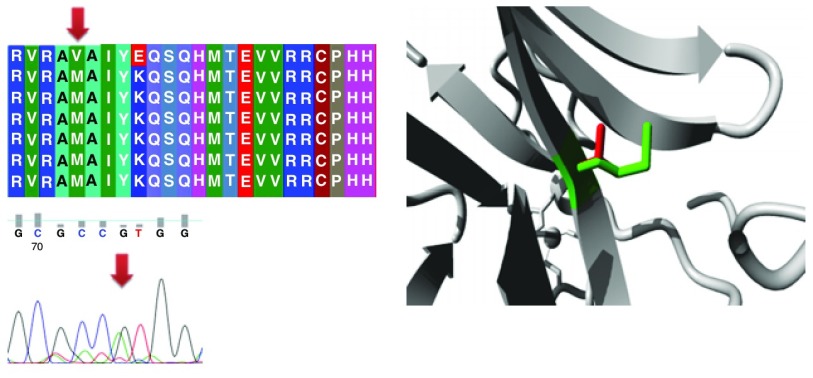
Mutation of a methionine into a valine at position 160.

**Figure 8.**
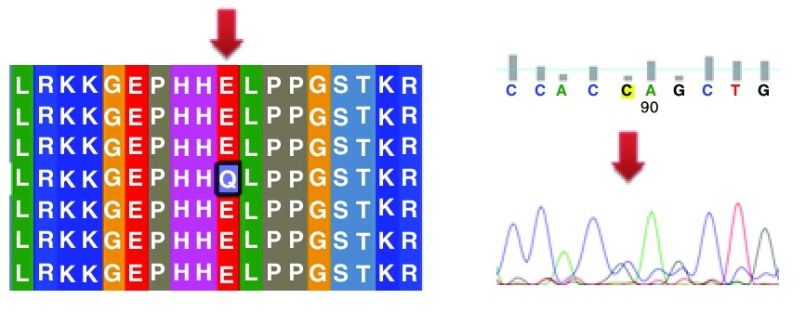
Mutation of glutamic acid into glutamine at position 298.

Patients information, clinical data, and histological findings of all patients were available in Excel format Click here for additional data file. Copyright: © 2018 Elfaki RM et al. 2018 Data associated with the article are available under the terms of the Creative Commons Zero "No rights reserved" data waiver (CC0 1.0 Public domain dedication).

## Discussion

Mutations of *TP53* gene can lead to loss of functional characteristics in tumor cells, as mutant *TP53* may not play the assigned role in repairing cellular machinery leading to a loss of normal function and, subsequently, cells with mutant gene may express uncontrolled replication which leads to accumulation of protein 53 ^13^.

There were 8 (40%) males with *TP53* gene mutations and 12 (60%) females. *TP53* mutations were found in 22.9% of esophageal SCCs and 14.3% of esophageal ACs in a study done by Zheng *et al*. ^35^ here the ratio was (34.9% vs. 57.1%) respectively. Sample size may be the main factor affecting the result.

Shi *et al.* ^36^ in Henan province, China showed that the p53 mutations were detected in 30 out of 43 (70%) SCC cases but in this study it represents 16 out of 43 (37.2%); this difference can be attributed to different factors e.g. geographical zone differences or type of exons studied as China has a high-incidence of esophageal carcinoma. Another study in Henan province, China conducted by Li-Ya ^15^ and his colleagues stated that p53 mutations were detected in 40.9% of their esophageal cancer specimens and this is compatible with our finding on mutations being detected in 20 out of 50 specimens of esophageal carcinomas which represent 40%.

In a study conducted by Zheng *et al*. ^35^ Qiqihar City, China, a higher mutation rate was found in SCC samples compared to AC samples (31.4% vs. 21.4%, respectively). And this partially differs from our result which found 4 out of 7 (57.1%) of adenocarcinoma patients had mutations which revealed higher mutation rate among AC samples, and this result may differ if had a larger sample size with more adenocarcinoma cases.

Exon 5 of the *TP53* gene was observed to be the most mutated exon among the other exons investigated here in this study, with 17/20 (85%) of all mutations detected found in exon 5, 2/20 (10%) in exon 6 and 1/20 (5%) in exon 8, while exon 7 showed no mutations. Uchino *et al.* ^37^ results for mutation distribution across exons was 39.3% in exon 5, 32.1% in exon 6, 17.9% in exon 7 and (10.7%) forexon 8 mutations. Which has partly agreed with our result in exon 5 having the higher mutation rate in addition to reasonable differences in other exons rate of mutation, and this can be attributed to the small sample size used in this study.

A total of 10% of all detected mutations were classified as neutral polymorphism, while 90% were considered disease-causing according to Mutation Taster, considered high rate of disease-causing mutations. This rate is lesser using other software due to different algorithms used by any one of them, their linked databases, and characteristics of the different software.

## Conclusions

Mutation of exon 5 in p53 gene were the most frequent in esophageal cancer. Genomic results have identified a high *TP53* mutation rate in esophageal Adenocarcinoma compared to squamous cell carcinoma.

## Ethical considerations

This study was approved by the Institutional Ethics Committee, Sudan University of Science and Technology (reference number for the ethical committee is DSR-IEC-13-05). Patients consent cannot be obtained because most of the patients were dead and the rest cannot be traced due to lack of contact data. Therefore all samples and medical data used in this study have been irreversibly anonymized to ensure patients privacy.

## Data availability

The data referenced by this article are under copyright with the following copyright statement: Copyright: © 2018 Elfaki RM et al.

Data associated with the article are available under the terms of the Creative Commons Zero "No rights reserved" data waiver (CC0 1.0 Public domain dedication). 

*TP53* sequences for this study has been submitted to Banklt NCBI and had been assigned the accession numbers MH366303 to MH366483. ** The sequences are available as 4 PopSet entries for exons 5–8:

Exon 5: 1472901613

Exon 6: 1472901713

Exon 7: 1472901809

Exon 8: 1472901897

*TP53* sequence results were submitted in a zipped file. These sequencing results as received from BGI Company (China) include 50 esophageal cancer patients in this study using the four sets of primers for exon 5, 6, 7 and 8. Sequencing files that needs to be viewed using FinchTV and Notepad file.

**Dataset 1:** Patients information, clinical data, and histological findings of all patients were available in Excel format. 10.5256/f1000research.15534.d219739 ^38^
