# Supplementary material for: Molecular and in-silico analysis of single nucleotide polymorphism targeting human TP53 gene exon 5-8 in Sudanese esophageal cancer patients
Source: F1000Res. 2018 Nov 2;7:1741. [Version 1] doi: 10.12688/f1000research.15534.1 (PMC6611135; doi:10.12688/f1000research.15534.1)
Supplement: Peer review history

# Peer review: Reviewer response for version 1

**DOI:** 10.5256/f1000research.16944.r50571

**Published:** 2019-07-16

**Authors:** Hideo Baba

**Affiliations:** 1 Department of Gastroenterological Surgery, Kumamoto University, Kumamoto, Japan

**Major revision**

 The authors evaluated single nucleotide polymorphisms (SNPs) in TP53 gene exon 5-8, and found that 37.2% of esophageal squamous cell carcinoma (SCC) samples harbored mutation, whereas 57.1% of esophageal adenocarcinoma (AC) samples harbored mutation. These result is interesting. However, this study lacks the information or explanation of SNPs in TP53 gene exon 5-8.

1. The authors need to discuss how SNPs in TP53 gene exon 5-8 influence developing of esophageal cancer.
2. The authors should mention the reason why the author focused on exon 5-8 in more detail.    
3. The author need to show the relationship between SNPs in TP53 gene exon 5-8 and clinicopathological features, including survival information.

I confirm that I have read this submission and believe that I have an appropriate level of expertise to confirm that it is of an acceptable scientific standard, however I have significant reservations, as outlined above.

# Peer review: Reviewer response for version 1

**DOI:** 10.5256/f1000research.16944.r50574

**Published:** 2019-07-01

**Authors:** Naoise Synnott

**Affiliations:** 1 National Cancer Institute, National Institutes of Health, Bethesda, MD, USA

In this article the authors carried out TP53 sequencing in 50 esophageal cancers, from the Sudan population. Sequencing identified 40% of samples had a TP53 mutation, with the majority of mutations occurring in the core domain in exon 5. This finding is consistent with the literature. A higher rate of TP53 mutations was found in Ac compared to SCC, however the sample numbers of each cancer type very greatly in this study. 

 Overall this is a well written article, with novel data on the Sudanese population, with some consistency with previous reports in other geographical locations. 

 I would suggest including data on the incidence, prevalence and mortality rate of SCC and AC in Sudan in the introduction (GLOBOCAN, 2018).

I confirm that I have read this submission and believe that I have an appropriate level of expertise to confirm that it is of an acceptable scientific standard.

# Peer review: Reviewer response for version 1

**DOI:** 10.5256/f1000research.16944.r40228

**Published:** 2018-11-14

**Authors:** Carlos Mario Muñetón Peña

**Affiliations:** 1 Medical Genetics Unit, Department of Pediatrics, Faculty of Medicine, University of Antioquia, Medellín, Colombia

In general it is a good paper, the results are shown in an orderly sequence, but some data of the results are confusing; the discussion is very concrete.

 The authors performed an interesting computational analysis with a battery of bioinformatics tools for the screen of the SNPs; these analyses are the strongest of the study.

 Other comments specific to the study:

1. According to the objective of the study, it would have been very interesting if the authors also studied the polymorphism ‘rs104252272 (Pro72Arg)’, identified in different populations and associated with risk to different types of tumors.
2. Regarding the classification of the variants I would recommend the authors use the ACMG nomenclature throughout the manuscript to homogenize the information, instead of using "deleterious SNPs, missense SNP”; by pathogenic variant, benign variant, VUS, etc.
3. The abstract does not mention the objective of the article or the size of the sample: In the results it appears "Six out of ten of TP53 gene mutations occurred in exon 5", but the authors describe that the mutated samples were 20 (40%) and not 10 mutations; exon 5 has more than six mutations. The authors should clarify this confusing data.
4. The authors should also describe in Table 2 the mutations identified using the international nomenclature to show exactly the position in which the substitution occurred and the type of mutation (example: c.595 G> T, GGA / TGA.)
5. Table 2 shows 18 mutations in exon 5, two in exon 6 and one in exon 8, but these data do not agree with the text, and the percentages are made from 10 mutations - these data must be reviewed to agree.
6. The description of the mutated SSC cases is not agreed in the manuscript - it is mentioned that there are 15 cases, but 16 cases are described (13 in exon 5, 2 in exon 6 and 1 in exon 8). In this situation, the authors must also present the most clear information.
7. Finally, in the discussion the authors compare their results of mutations in the TP53 gene only with studies from China - it would be very interesting if they would also compare them with similar studies carried out in other populations (European, American, Hispanic American, etc.).

I confirm that I have read this submission and believe that I have an appropriate level of expertise to confirm that it is of an acceptable scientific standard.
